# Genetic tumor syndromes – It’s time for action

**DOI:** 10.1515/medgen-2025-2042

**Published:** 2025-11-08

**Authors:** Evelin Schröck, Reiner Siebert

**Affiliations:** University Hospital Carl Gustav Carus at TU Dresden Institute of Clinical Genetics Fetscherstr. 74 01307 Dresden Germany; Ulm University & Ulm University Medical Centre Institute of Human Genetics Albert-Einstein-Allee 11 89081 Ulm Germany

Genetic tumor syndromes definitely deserve more attention by human geneticists and other medical professionals of a broad spectrum of clinical disciplines than they traditionally have received. Data from large sequencing studies point to the fact, that around 10 % of all patients diagnosed with a tumor carry a (likely) pathogenic germline variant. Remarkably, this number does hold true across all age groups and include at least a third of patients in which classical clinical criteria for diagnosing a genetic tumor syndrome including a family history of cancer are not fulfilled. The incidence of germline pathogenic variants does vary between tumor entities. Nevertheless, we yet have an incomplete recognition both on rare tumors with a high rate of patients with germline variants, such as uterine leiomyosarcomas, as well as on common cancers like pancreatic or prostate carcinomas, with a substantial number of individuals carrying a germline predisposition. Moreover, as impressively indicated by patients with more than one tumor, which are more likely to carry a pathogenic germline variant in a cancer predisposition gene, genetic tumor syndromes do not respect the borders between clinical disciplines, independent of whether they are defined by age, sex, organ system or way of treatment. The following articles therefore aim to draw the attention to the topic of genetic tumor syndromes and the many patients who are still being overlooked.

The increasing clinical importance of cancer predisposition in oncology and haematology is highlighted by the fact, that for the first time a WHO classification of genetic tumor syndromes has been recently developed. Siebert and Foulkes review the current status of this classification, which applies a molecular mechanism- and pathway-based hierarchical system according to Linnean principles [1]. By this approach, it parallels up-front pathway-driven treatment approaches in personalized onocology. The classification also includes many ultrarare genetic tumor syndromes which are not so familiar to many health professionals as compared to e.g. Li-Fraumeni syndrome and von Hippel-Lindau disease. Therefore, such ultrarare genetic tumor syndromes are prone to be overlooked. Thus, especially these conditions are reviewed by Genuardi and colleagues [2]. On the opposite side of the spectrum are common genetic tumor syndromes, which are well-known to many geneticists and clinicians but nevertheless also at risk to be overlooked, here because in a substantial fraction of individuals they are caused by pathogenic variants presenting in mosaic forms. As Boros et al. summarize, the probability of such mosacisms depending on the type of genetic tumor syndrome and affected gene can be substantial reaching e.g. 10 % and more of cases [3]. These observations render dedicated searches for mosaicism according to proposed algorithms an important complementary approach to whole genome sequencing in patients with typical features.

The second part of this issue is dedicated to some genetic tumor syndromes which pose particular challenges to geneticists and clinicians. Here, as described by Hoogerbrugge and colleagues, *CHEK2*-associated tumour predisposition are a prominent example [4]. The authors show how the variable penetrance of a moderate-risk genes like *CHEK2*, where lifetime cancer risks range from 15 % to 55 % depending on multiple factors, demands personalized risk assessment beyond traditional paradigms. Turning to still one of the deadliest cancers, William et al. provide an overview on the genetic predisposition to pancreatic cancer [5]. They show that some cohorts report germline variant rates exceeding 20 % in pancreatic cancer, with genes like *BRIP1* approaching the frequency of established predisposition genes.

The third part of this issue is dedicated to targeted therapy and therapy risk in genetic tumor syndromes. Distel and colleagues review the issue of radiation sensitivity in genetic tumour syndromes and how to test for them [6]. They show, that e.g. treatment considerations around radiation sensitivity in *TP53*-associated syndromes require sophisticated genetic input. Finally, Jahn and colleagues provide an overview on targeted therapy in patients with genetic tumor syndromes [7].

The data highlighted in this issues show that the clinical implications of identifying a germline pathogenic variant are profound. For patients, a genetic tumor syndrome diagnosis will result in measures such as surveillance procedures, which could potentially lead to earlier tumor detection and improved survival. For families, cascade testing can identify at-risk relatives, enabling preventive interventions such as risk-reducing surgeries or chemoprevention. Furthermore, germline findings can influence treatment decisions, particularly in metastatic settings, where therapies targeting specific molecular vulnerabilities (e.g., PARP inhibitors in BRCA-related cancers) are now the standard of care.

The articles moreover indicate, that now is the time to increase our efforts and open up the clinical management opportunities available to breast and ovarian hereditary cancer patients to all patients and families at risk for a genetic tumor syndrome. Once considered rare and largely academic curiosities, these syndromes are now at the forefront of human genetics and clinical oncology, transforming our understanding of cancer etiology, risk assessment, and prevention. As genomic technologies advance and our knowledge deepens, the recognition and management of genetic tumor syndromes must evolve from reactive diagnosis to proactive, precision-based strategies.

The future of genetic tumor syndromes lies in multidisciplinary strategies including clinical geneticists, oncologists, surgeons, counselors, and bioinformaticians. Artificial intelligence and machine learning may soon help to summarize existing knowledge and predict potential mechanisms. Additionally, research into modifier genes and epigenetic factors could clarify why some mutation carriers develop cancer while others do not.

In conclusion, genetic tumor syndromes are now a cornerstone of modern clinical genetics and oncology. They exemplify the power of genomics to transform cancer care, from prevention to treatment. As we continue to decode the genetic architecture of cancer susceptibility, our responsibility grows: to ensure equitable access to testing, to provide compassionate and informed counseling, to detect cancer earlier and to prevent cancer, one inherited risk at a time.
